# Supplementary material for: HIV, syphilis and sexual-risk behaviours’ prevalence among in-and out-of-school adolescent girls and young women in Zambia: A cross-sectional survey study
Source: PLoS One. 2024 Jun 5;19(6):e0294545. doi: 10.1371/journal.pone.0294545 (PMC11152284; doi:10.1371/journal.pone.0294545)
Supplement: Attachment — Submitted filename: Response to reviewers.doc [file pone.0294545.s001.doc]

## THE UNIVERSITY OF ZAMBIA

**SCHOOL OF PUBLIC HEALTH**

**Department of Epidemiology and Biostatistics**

Telephone: 252641, P.O. BOX 50110

**E-mail**: pmuzho@hotmail.com	Lusaka, Zambia

DATE: 04/03/2024

==============================================================================================

Dear Editor (Plos One journal),

**RE: Response to the reviewers for the research article entitled:**

**HIV, Syphilis and Sexual-risk behaviours’ prevalence among in-and out-of-school adolescent girls and young women in Zambia: A cross-sectional survey study.**

I write to respond to the valuable points raised by the reviewers. The format of the response will be such that; we will state the reviewer’s comment and our response followed by any changes we will have made in bold italics.

**Comment from reviewer**:

We note that you have provided funding information that is currently declared in your Funding Statement. However, funding information should not appear in the Acknowledgments section or other areas of your manuscript. We will only publish funding information present in the Funding Statement section of the online submission form.

Please remove any funding-related text from the manuscript and let us know how you would like to update your Funding Statement. Currently, your Funding Statement reads as follows:

“This study was funded by the Global Fund. The Zambia AGYW program is part of the Global Fund Strategy (2017-2022) to reduce new HIV infections among AGYW by 58% by 2022 in13 sub-Saharan African countries including Botswana, Cameroon, Kenya, Lesotho, Malawi, Mozambique, Namibia, South Africa, Eswatini, Tanzania, Uganda, Zambia and Zimbabwe. However, the funders played no part in writing of this manuscript, any errors or mistakes are thoroughly the responsibility of the authors.”

**Author’s response:**

***Thank you to the reviewer for the above comment, however, when we read through the acknowledgement, we don’t see the said funding statement in the acknowledgement, but we agree that the funding statement quoted above is correct, but is declared in the right place, not as suggested that it is in theour acknowledgement statement reproduced below:***

“*We would like to express our heartfelt gratitude to all the individuals and institutions who supported and contributed towards the implementation of this project. Without their valuable support, this project would not have been feasible. We are especially grateful to the Ministry of Health and Global Fund through the Prevention of HIV, TB, and Malaria for providing financial and technical support for this project. We are also further grateful to the Ministry of Health Leadership through the Adolescent Health Unit for their oversight and guidance throughout the study. Their expertise and support were vital in ensuring that the research adhered to the highest standards of ethics and safety.*

*We would also like to extend our thanks to CIDRZ for their significant contribution to the success of this project. Their facilitation of testing of samples and maintaining quality controls were critical in ensuring the accuracy and reliability of the data.*

*We also extend our sincere thanks to the Ministry of Health, Education Government Departments, Non-Governmental Organisations, Community Radio Stations, and Zambia National Broadcasting Corporation for their support during the data collection process. We also extend our appreciation to the District Health Promotion and Adolescent Health Officers for their tireless efforts towards sensitization and data collection. Their dedication and commitment to promoting HIV testing and counselling for adolescent girls and young women in Zambia is commendable.*

*Additionally, we extend our gratitude to the research assistants, laboratory scientists, and counsellors for their valuable contributions to this project. Their hard work and dedication towards data collection and analysis were crucial to the success of this research.*

*Furthermore, we would also like to extend our gratitude to all the investigators who played a crucial role in this study. Their expertise, guidance, and support were vital to the success of this research project. Finally, we would also like to express our gratitude to the project management team for their tireless efforts in overseeing the day-to-day activities of this project. Their efficient planning and execution of the project ensured that the research was completed within the stipulated timeframe and budget.*

*Finally, we would like to acknowledge the traditional, religious leaders and parents in the various study sites for their support and guidance. Their involvement was instrumental in ensuring that the research was culturally sensitive and relevant to the local community. Special thanks to all those who agreed to participate in the study, as without them, this report could not have been possible*”.

**Comment from reviewer (3rd comment)**

3. In the online submission form you indicate that your data is not available for proprietary reasons and have provided a contact point for accessing this data. Please note that your current contact point is a co-author on this manuscript. According to our Data Policy, the contact point must not be an author on the manuscript and must be an institutional contact, ideally not an individual. Please revise your data statement to a non-author institutional point of contact, such as a data access or ethics committee, and send this to us via return email. Please also include contact information for the third party organization, and please include the full citation of where the data can be found.

**Author’s response:**

***Thank you so much for the above, below is the contact person we are suggesting.***

*We are suggesting Smart Zambia, a government agency that will have access to the MoH server for data and the contact will be the Director ICT Mr Andrew Kashoka; email* [*andrewkashoka@gmail.com*](mailto:andrewkashoka@gmail.com)*.*

**Comment from the reviewer (4th comment):**

4. We note that Figure 1 in your submission contain [map/satellite] images which may be copyrighted. All PLOS content is published under the Creative Commons Attribution License (CC BY 4.0), which means that the manuscript, images, and Supporting Information files will be freely available online, and any third party is permitted to access, download, copy, distribute, and use these materials in any way, even commercially, with proper attribution. For these reasons, we cannot publish previously copyrighted maps or satellite images created using proprietary data, such as Google software (Google Maps, Street View, and Earth). For more information, see our copyright guidelines: <http://journals.plos.org/plosone/s/licenses-and-copyright>.

We require you to either (1) present written permission from the copyright holder to publish these figures specifically under the CC BY 4.0 license, or (2) remove the figures from your submission:

1. You may seek permission from the original copyright holder of Figure 1 to publish the content specifically under the CC BY 4.0 license.

We recommend that you contact the original copyright holder with the Content Permission Form (<http://journals.plos.org/plosone/s/file?id=7c09/content-permission-form.pdf>) and the following text:

**Response to the reviewer (4th comment).**

***Thanks for the above comment and request. We want to make mention that Figure 1 and indeed all other figures in the manuscript, we did not copy them from any source, we created them using freely available software, for other Figure 2, we used well known statistical software Stata whose license is legally owned.***

***The map was created using QGIS 3.28.13 and basemap data used was downloaded from*** [***Zambia - Subnational Administrative Boundaries - Humanitarian Data Exchange (humdata.org)***](https://emea01.safelinks.protection.outlook.com/?url=https%3A%2F%2Fdata.humdata.org%2Fdataset%2Fcod-ab-zmb&data=05|02||de99af0fbd694d47c9ae08dc3d9c0401|84df9e7fe9f640afb435aaaaaaaaaaaa|1|0|638452987525968214|Unknown|TWFpbGZsb3d8eyJWIjoiMC4wLjAwMDAiLCJQIjoiV2luMzIiLCJBTiI6Ik1haWwiLCJXVCI6Mn0%3D|0|||&sdata=UP2K6Uqv14RRQVnNPG4TagDbYMQ%2FKWcTbnB1K9YRppI%3D&reserved=0)***. The Humanitarian data exchange has the "Creative Commons Attribution International(CC BY)" license. This license permits us to share and adapt maps with necessary attributes. Please see link to the licence:*** [***Data Licenses - Humanitarian Data Exchange (humdata.org)***](https://emea01.safelinks.protection.outlook.com/?url=https%3A%2F%2Fdata.humdata.org%2Ffaqs%2Flicenses&data=05|02||de99af0fbd694d47c9ae08dc3d9c0401|84df9e7fe9f640afb435aaaaaaaaaaaa|1|0|638452987525978089|Unknown|TWFpbGZsb3d8eyJWIjoiMC4wLjAwMDAiLCJQIjoiV2luMzIiLCJBTiI6Ik1haWwiLCJXVCI6Mn0%3D|0|||&sdata=XaZoaTIpRNXQuNK8%2BTs25paLtsHMbDM7VvOLMwosS%2FQ%3D&reserved=0)***.***

**Reviewer’s comment (5th comment).**

5. Please include a separate caption for each figure in your manuscript.

**Response from the author’s.**

We only had two figures, and for each, a separate caption is indicated just below the figure.

**Comment from the reviewer (1^st^ comment)**:

1. Why do AGYW in Zambia and other developing nations have a much higher incidence of HIV when compared to the men (5.6%vs 1.8%)

**Response from the author’s:**

***Thanks to the reviewer to the above query, we are inserting the following paragraphs in the discussion just below the section entitled “Vulnerability levels of households and individual AGYW” : .***

*“The vulnerabilities of AGYW observed also contributes to the observed high prevalence in AGYW compared to their men counterpart. In addition, girls' intimate relationships and sexual debut is earlier than their male peers; and secondly, they have partners older than them, some of whom emanate from age groups/brackets with higher HIV incidence. The power dynamics in these relationships usually leave the female folk vulnerable, especially amidst questionable condom use and/or its absence and lack of other personalized, and female tailored HIV prevention interventions such as the Cabotegravir injectable and Dapivirine ring, respectively. Both of which are only being introduced now. There is also a biological part which makes it easier for HIV acquisition in these young females. Of course, in our setting, there is the issue of Sexual Gender Based Violence (SGBV) which is a big problem in Zambia. Our study showed that all these factors contributed to the fact that HIV prevalence was high among AGYW compared to men”.*

**Reviewer’s comment: (2nd comment)**

2. The authors have mentioned the seroprevalence of HIV and Syphilis in the groups district wise. It would be nice to know how many were positive for both HIV and Syphilis , if this data is available.

**Response to the reviewer:**

***on page 46 in the manuscript, we report the following paragraph, it appears like the reviewer did not see this, this is giving the prevalence for all and for in and out of school adolescents:***

“*Out of 12, 457 participants the overall prevalence of HIV was 466 (3.7%) 95% CI: 3.4 – 4.1). The weighted prevalence of HIV was higher in the out-of-school compared to the compared to the in-school participants (5.5% vs 2.0%), Similarly, the prevalence of syphilis was higher in the out-of-school than the in-school participants (4.1% vs 1.5%). When the HIV prevalence was stratified by age group, highest prevalence was reported among those aged 20 – 24 years (5.8%) while the least was among those aged 10 – 14 years (0.7%).*

*Likewise, highest prevalence of syphilis was reported among participants aged 20 – 24 years (4.7%) and lowest was among those aged 10 – 14 years (0.5%) as shown in Table 9*”.

**Comment from the reviewer:**

3. One of the objectives of the study as mentioned in Introduction was to determine the incidence of GBV in these groups of AGYW. However nothing is mentioned about GBV in the discussion.

**Response to the reviewer:**

***We agree with the above comment. We did not make it clear that the main findings on GBV are in the qualitative paper, but in this quantitative paper, we do have a small section on GBV which we presume the reviewer did not see, we high light this section in the manuscript with track changes and we paste what that section says below:***

***Linkage to care among AGYWs confirmed to be HIV or Syphilis Positive, and AGYW reporting Gender-based Violence.***

*All AGYWs confirmed HIV-positive were referred to the nearest health facility for enrolment into HIV care as per existing enrolment guidelines. In addition, those who tested HIV negative but were syphilis positive were referred to the nearest health facility for further management. Individuals that report gender-based violence were referred to the existing gender-based violence programs in the community for better management.*

**Comment from the reviewer:**

“4. What are the SRH services (or lack of it) available in Zambia and how this can be improved as SRH services availability was also one of the objectives of the study”.

**Response to the reviewer:**

**Thanks once again for this useful suggestion, in the discussion we add the following paragraph:**

**Sexual Reproductive Services in Zambia:**

***“The SRH services available in Zambia are: Family planning  (contraceptive) services, antenatal, postnatal, delivery, safe and post abortion care, cervical cancer screening, HIV testing and treatment services, Human Papilloma Virus Vaccination for pre and adolescent girls aged between 9 and 14, menstrual hygiene and management. These services are available and accessible at different levels of the health care system from primary to tertiary level and ranging from information only, counselling, and actual services.***

***However, the SRH services availability can be improved by ensuring availability of SRH commodities and equipment, trained personnel, and right infrastructure for provision of comprehensive SRH services”.***

**Comment from the reviewer**:

5. What suggestions do the authors have to improve the situation of AGYW in Zambia and decrease their vulnerability.

**Response to the Reviewer:**

***Thanks for the above comment, we have now inserted a section giving recommendation as shown below:***

**“*Recommendations***

*These findings support the view that there is a need for interventions that can help to keep girls in school, provide training for teachers on how to deliver comprehensive sexual education (CSE) effectively, engage with community leaders and parents to build support for CSE implementation and Increase funding and resources for implementing CSE programmes including providing materials and equipment for teaching. Among those AGYW already out of school, there is a need for unique interventions to reduce risk taking behaviours such as improving on their ability to negotiate for safer sex, and reduce their vulnerability to the risk of HIV and other STIs”.*

Yours Sincerely,

Professor Patrick Musonda

(Lead and corresponding author )
